# Nesting ecology of hawksbill turtles, *Eretmochelys imbricata*, in an extreme environmental setting

**DOI:** 10.1371/journal.pone.0203257

**Published:** 2018-09-07

**Authors:** Mark Chatting, David Smyth, Ibrahim Al-Maslamani, Jeffrey Obbard, Mehsin Al-Ansi, Shafeeq Hamza, Salman Fahad Al-Mohanady, Ali Jassim Al-Kuwari, Christopher D. Marshall

**Affiliations:** 1 Environmental Science Center, Qatar University, Doha, Qatar; 2 College of Arts and Sciences, Qatar University, Doha, Qatar; 3 Ras Laffan Industrial City, Doha, Qatar; 4 Ministry of Municipalities and Environment, Doha, Qatar; 5 Department of Marine Biology, Texas A&M University at Galveston, Galveston, Texas, United States of America; University of Sao Paulo, BRAZIL

## Abstract

Relatively few details of hawksbill turtle (*Eretmochelys imbricata*) nesting ecology exist within the Arabian Gulf. Moreover, little is known about how their nesting dynamics compare to nesting populations throughout the rest of the world. Due to the extreme environmental setting, nesting ecology of hawksbills in the Arabian Gulf is of significant interest to researchers and conservationists. The current research reports on a long-term tagging and monitoring program undertaken at Fuwairit beach, Qatar. To investigate nesting behavior, site surveys and tagging were employed from 2010 to 2016. Presence of nests and clutch sizes were confirmed by excavation. Over the entire study period, nesting hawksbills had a mean curved carapace length of 70.8 cm (SD±2.8). A total 187 nests were confirmed, which contained a mean 78.9 eggs per clutch (SD±17.1), over an annual nesting season that lasted an average of 52.2 days (SD±6.3) from the start of April to the start of June. Meta-analysis with other global regions showed these characteristics to be significantly reduced when compared to nesting hawksbills from other populations. Meteorological data analysis showed air temperatures in the Arabian Gulf to increase on average 13.2°C (SD±0.26) from start to the end of nesting annually, which is significantly greater than other global nesting regions. Their smaller body size and reduced fecundity coupled with the extreme change in ambient air temperatures support the hypothesis that hawksbills in the region are more at risk than the already critically endangered hawksbill populations elsewhere in the world.

## Introduction

Hawksbill sea turtles (*E*. *imbricata*), listed as critically endangered by the IUCN (International Union for the Conservation of Nature), have a well-documented circum-global distribution [1–7]. Nesting aggregations have been recorded throughout tropical and, to a lesser extent, subtropical waters of the Atlantic, Indian, and Pacific Oceans [1,3,8–10]. Research throughout these locations has shown variations in nesting ecology which may be driven by regional environmental differences [3,4,11,12]. Despite this, little is known about the nesting dynamics of hawksbills in the Arabian Gulf, an extreme sub-tropical environment. Hawksbills in the region can offer a greater understanding of how the species’ reproductive ecology functions under environmental stress. Recent evidence has suggested that hawksbills in the region are smaller and lay smaller clutches [13,14], however, to date, limited work has focused on comparing nesting dynamics of these populations to their tropical counterparts. Moreover, no work has used statistical analysis to confirm whether apparent differences reproductive ecology are significant.

The Arabian Gulf has repeatedly been reported to be an extreme marine environment [13,15–22]. Summer sea surface temperatures (SST) regularly reach 35°C which is close to the physiological tolerances of many marine organisms [16,17]. It is a semi enclosed basin, characterized by shallow depths (mean = 35 m; max = 160 m) which when combined with high SST and high evaporation rate, up to 2 m yr^-1^, leads to hypersaline water (mean = 40; max = 70) [23]. Moreover, because of its location in the sub-tropics, experiences significant temperature changes from summer to winter (air temperatures ranging from 10°C in the winter to over 50°C in summer) [24,25]. This wide range of temperature regimes puts considerable pressure on coastal habitats. Coral communities in the region are dominated by heat tolerant species [20]. Under this stress, coastal habitats, suffer from reduced productivity compared to more favourable, tropical settings and many marine organisms are prone to dwarfism [24–26]. Hawksbill nesting dynamics are still not well understood under these conditions.

Global variations in nesting ecology have been shown to drive regional fecundity [3,4,27,28]. Variation in nesting ecology include variation in nesting season length, clutch frequency, remigration intervals, size of nesting females and clutch size. All of these factors influence regional differences in nesting dynamics [1,2,4,29,30]. Nesting season lengths vary from 5 months to year round in many parts of the world [2,10,28]. The mean Curved Carapace Length (CCL) of nesting females range from 80 to 95 cm [5,7,9,31]. Body size (CCL) is significantly correlated with clutch sizes from 155 eggs per clutch in the Caribbean, to 182 eggs per clutch in the Indian Ocean, to 124 eggs per clutch in Australia [4,32,33]. Baseline data such as these provides an important starting point for the conservation of hawksbill turtles, however, the majority of these studies have been performed in similar environmental settings in the tropics. Because of this, little is known about hawksbill nesting dynamics outside the tropics under extreme environmental conditions or how these regional variations correlate with environmental parameters.

Hawksbills in the Arabian Gulf have a 3 month nesting season from April to June, much shorter than the 6 to 12 months reported elsewhere [2,28,34,35]. However, it is not known why nesting seasons vary widely between biogeographic regions. Little work has investigated the environmental conditions before and after nesting periods. Air temperatures in particular, which have been shown to be strongly correlated with nest temperatures and subsequently hatchling sex ratios [36] may play an important role in limiting nesting season lengths in the Arabian Gulf. In addition, the largest reproductive female previously recorded in the region had a CCL of 74 cm, which is considerably smaller than the 80 cm to 95 cm reported from other global populations [4,33–35]. Even in the Arabian Sea, a geographically close, but separate nesting population, where environmental conditions are less extreme, CCL’s of hawksbills reach up to 90cm [13,37,38].

Despite these recent investigations into hawksbill nesting in the Arabian Gulf, little is known about how their nesting dynamics compare with other global populations [34,35,39,40]. Certain populations of this critically endangered species may be more vulnerable than others due to their natural setting. A greater understanding of hawksbills’ reproductive capabilities under environmental stress will aid conservation and management efforts globally. The aim of this study was to formally document the findings of a long term monitoring project on hawksbill nesting in Qatar and compare it to other global locations. We also wanted to test the hypothesis that hawksbills from the region are smaller in size, have smaller clutches and experience the greatest change in air temperature from before to after annual nesting periods.

## Materials and methods

### Study site

**Fig 1:**
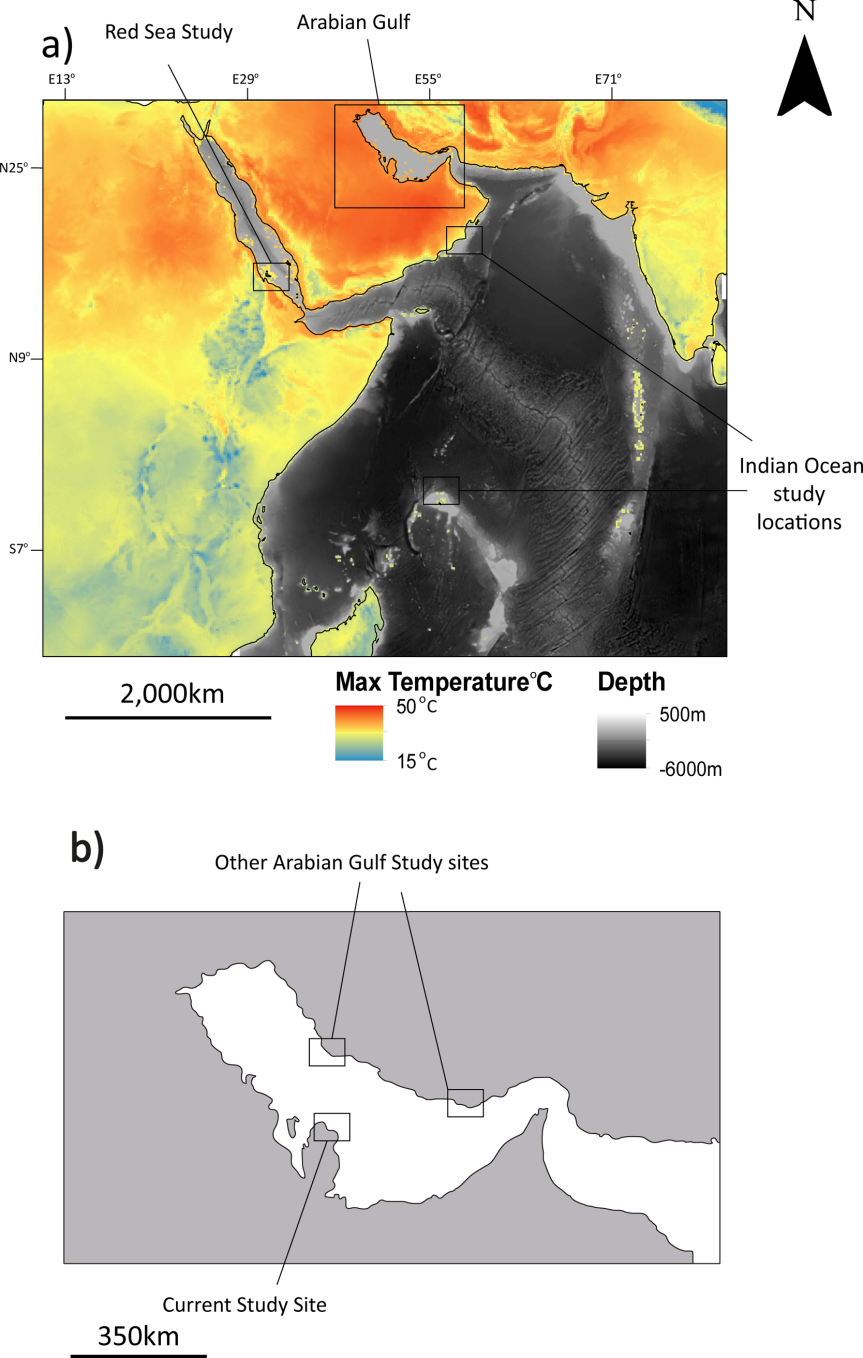
**a)** Bathymetry and maximum summer temperatures of the western Indian Ocean and locations of previous hawksbill nesting ecology studies [3,12]; and **b)** the Arabian Gulf also with locations of previous studies.

To ensure the ethical treatment of hawksbills, all interactions were minimized and monitored by the Ministry of Municipalities and Environment (MME). Prior to the commencement of working with hawksbills, MME approval was sought and granted to work directly with the animal and on their nesting grounds in Qatar. All technicians that had contact with hawksbill turtles were trained to Qatar University standards approved by the MME. Nesting site surveys were conducted in Qatar in the Arabian Gulf (Fig 1A). Nesting in the region occurs from winter to summer, typically from April to June. Surveys were conducted on Fuwairit beach, north east Qatar (N 26.032261^o^, E 51.373899^o^, Fig 1B). The beach is a sand bar 2.4 km in length covering an area of 0.28 km^2^. The beaches in the north east of Qatar are nesting sites for hawksbills, however, the present study was only conducted on the Fuwairit site.

### Survey effort

Access to Fuwairit beach was granted by the Ministry of Municipalities and Environment (MME). Nightly surveys were conducted on Fuwairit beach from 2010 to 2016. Each year, the start and end of nest surveying was based on turtle sightings by local MME rangers who patrol the site regularly throughout the year. All surveys began at sunset and finished at sunrise. The number of nights between the first and last nests encountered was calculated as the nesting season length.

### Flipper tagging

All nesting hawksbills encountered were tagged to identify individual nesters and remove duplication of individuals in later data analysis. Protocol required the turtle to be inspected for current tags or evidence of previous tagging before a new tag was fitted. All turtles encountered were tagged with a unique numbered ID Inconel tag [4]. Inconel tags weighing 3.19 g numbered consecutively were used. Trained technicians attached two tags, one to the right and left front flippers, to each observed turtle. All tags attached or encountered on a turtle were recorded with a corresponding GPS location and date. Tagging data was used to estimate clutch frequencies and internesting intervals. These data require more complete survey coverage and more sites to accurately estimate these parameters however, best estimates are reported in this study with levels of uncertainty to add to current datasets in the region The tagging program is a long term project and it is envisaged that more comprehensive datasets of interesting intervals and clutch frequencies will be obtained in the future. Curved Carapace Length (CCL) was measured from the shell notch to tip, and Curved Carapace Width (CCW) around the widest part of the shell were collected from each turtle encountered.

### Nesting

The total number of egg clutches were confirmed by excavation and reburial and were recorded each year. Turtle tracks were examined and recorded, if considered a probable nest it was excavated to determine presence of a clutch. Clutches were relocated to a protected hatchery from 2010 to 2014 and in 2016 as part of a wider conservation project. During this process, eggs were counted to determine clutch sizes. In 2015, clutch size was determined by counting eggs after the presence of a clutch was confirmed by excavation, then left in situ. Internesting intervals were calculated, in days, using tagging data collected from turtle observations from 2010 to 2016. An observed Internesting Interval (OII) was calculated, if a turtle visited more than once in a season, by subtracting the most recent observed nesting date from its previous confirmed nesting date. Observed Internesting Intervals < 7 d and >20 d were excluded on the assumption that the previous visit represented a failed nesting attempt or a missed nesting event respectively [3,28]. The Observed Clutch Frequency (OCF) was calculated by the number of confirmed nests a female laid in a season. Estimated Clutch Frequency (ECF) was calculated as ECF = 1 + (difference in days between first and last nesting / median OII) [28,41]. If an individual only nested once in the season the ECF was recorded as 1.0. ECF is regarded as a more accurate measure of clutch frequency from its recognition of varying survey effort and is a way of accounting for missed nesting events. As hawksbill clutches in the region have been shown to contain small yolkless eggs [39], only bigger, viable eggs were included in the clutch size count.

### Global nesting meta-analysis

To compare nesting ecology in the Arabian Gulf with other global regions, available raw data were collected from previously published work (S1 Table). Studies describing aspects of nesting dynamics described in the present study were searched. Specifically, references that described hawksbill CCL, CCW and clutch size from different global locations were used. There are numerous studies that describe CCL, CCW and clutch size throughout the world, however, only studies that provided raw data were included in the analysis. When carapace length was reported as Straight Carapace Length (SCL) it was converted to CCL using hawksbill specific allometric equations [42]. Only previously published studies or governmental reports were used. Where data were available, these studies were then grouped into regions (Asia-Pacific, Indian Ocean, Caribbean, West Atlantic) to allow for later statistical comparison with Arabian Gulf data.

### Meteorological data analysis

Meteorological data was gathered to compare air temperature before and after nesting seasons to investigate changes in air temperatures with other global locations. Air temperature data from the NOAA National Center for Environmental Prediction (NCEP) reanalysis project was used in the analysis [43]. Spatial and temporal resolution of downloaded meteorological datasets were 1^o^ and 1 month respectively. Downloaded data sets were then interpolated to the coordinates of known global hawksbill nesting grounds for the start and end of reported nesting seasons. The months of peak nesting activity were used when nesting was reported year round. Change in temperature from the start to the end of each season for all regions were calculated compared.

### Data analysis

Statistical analysis was carried out to test whether nesting dynamics in Qatar varied between years. A one-way Analysis of Variance (ANOVA) was used to compare years. The dependent variables used in this analysis were: CCL, CCW and clutch size. Linear regression was used to investigate the relationship CCL and CCW had with clutch size. Because of high collinearity between CCL and CCW multiple regression was not used in the analysis. Using global data, ANOVA was performed each on CCL, clutch size and change in air temperature with region as a factor. Log transformations were applied when data did not meet ANOVA test assumptions. When significant difference was detected, Tukey honest significance difference tests were used to see where differences were, if any were detected by ANOVAs. Linear regression was used to compare global locations’ CCL with clutch size. Tests were performed using R 3.1.2 software.

## Results

### Tagging

Between 2010–2016, hawksbill nesting in Qatar occurred from April-June with a mean nesting season duration of 52.2 days (SD ± 6.3) from the start of April to the start of June (S2 Table). During this period, 90 nesting individuals were tagged and measured (S3 Table). Mean CCL over the study period was 70.8 cm (SD±2.8) and CCW was 64.9 cm (SD±2.8). Curved Carapace Length and CCW showed no significant variation with year (CCL ANOVA: F _1,89_ = 1.819,p = 0.18 and ANOVA: F _1,89_ = 0.027,p = 0.87 respectively).

### Nesting

Throughout the study period, there were 38 instances of individuals returning to nest within the same season by 27 individuals. A mean OII of 14.8 days (SD± 1.9) was observed, which coincided with a mean OCF and ECF of 1.4 (SD± 0.7) and 1.5 (SD± 0.9), respectively (S1 Fig). The total number of egg clutches confirmed from 2010 to 2016 was 187. The mean number of egg clutches laid per year was 29.4 (SD±12.9) and ranged from 15 in 2013 to 55 in 2010. The mean number of eggs per clutch was 78.9 (SD±17.1). The largest clutch size had 128 eggs in 2014 and the smallest had 30 eggs in 2015. No significant variation in clutch size was detected between years (ANOVA: F _1,124_ = 2.34,p = 0.12). There was a weak relationship between clutch size and CCL (Regression: F_1,85_ = 7.20; R^2^ = 0.07; p<0.01) and CCW (Regression: F_1,85_ = 6.62; R^2^ = 0.06; p = 0.01).

### Global nesting meta-analysis

**Fig 2:**
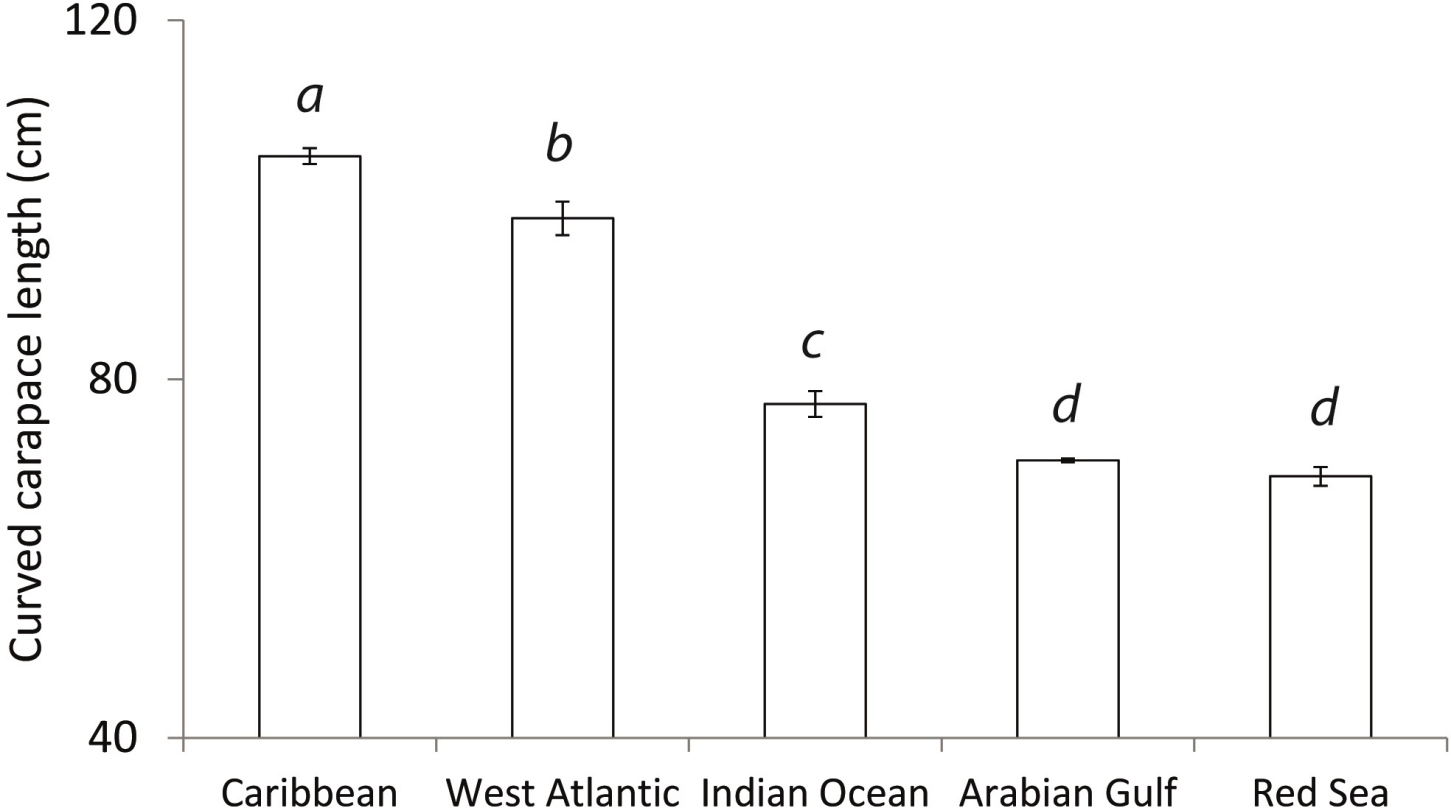
Comparison of CCL from different regions. Significant difference found between regions (ANOVA: F_4,470:_ = 989.6; p<0.01). Data for the Arabian Gulf was from the present study, Pilcher et al. (2014) and Pazira et al. (2016); raw data from the Caribbean was collected from Xavier et al. (2006); Ross (1980) provided data from the Indian Ocean; Red Sea data was from Hirth and Abdel Latif (1980) and West Atlantic data was from Marcovaldi et al. (1999). Regions with significantly different CCL’s are represented by superscript letters above the bars.

**Fig 3:**
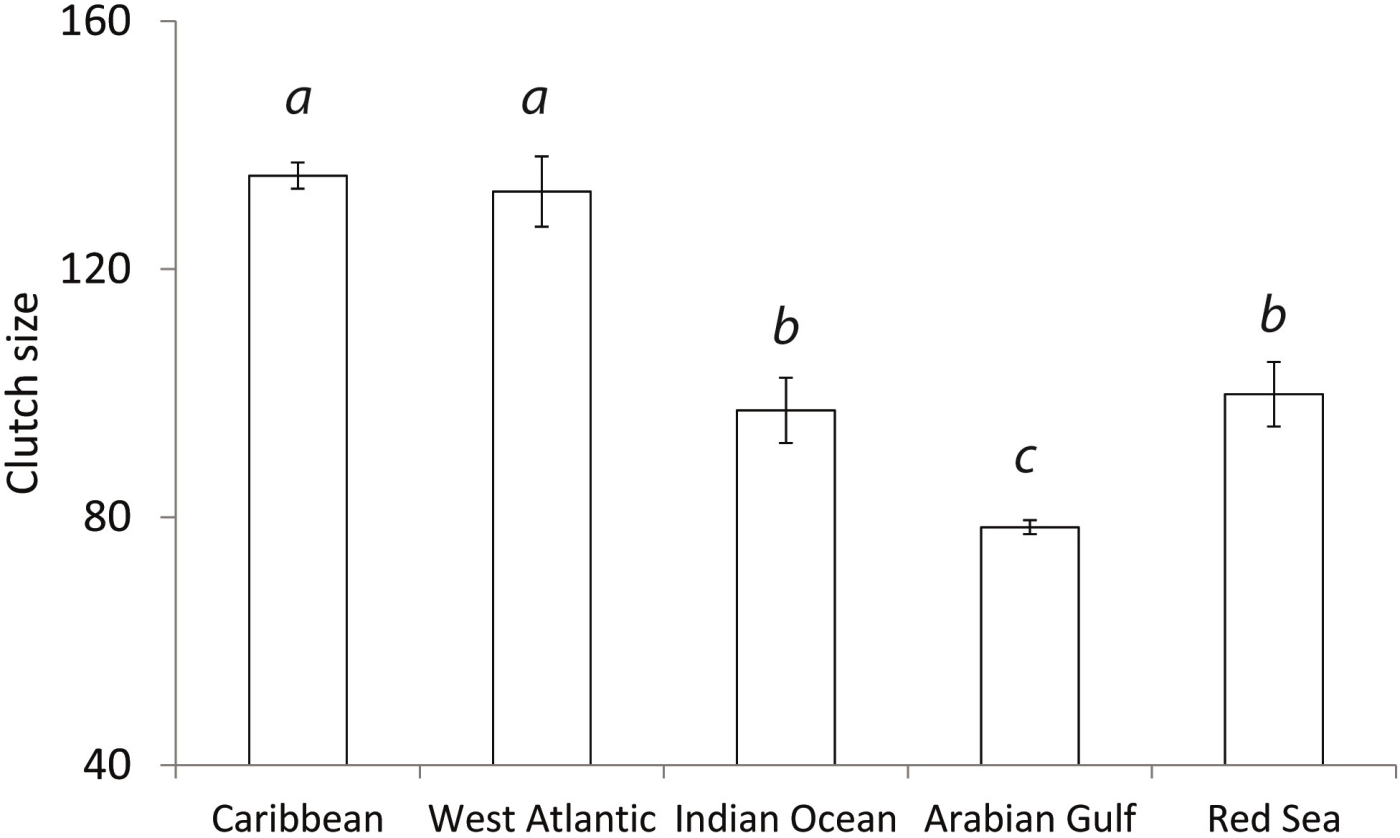
Comparison of clutch sizes from different regions. Significant difference found between regions (ANOVA: F_4,412_ = 156.2; p<0.01). Data for the Arabian Gulf was from the present study and Pazira et al. (2016); raw data from the Caribbean was collected from Xavier et al. (2006); Ross (1980) provided data from the Indian Ocean; Red Sea data was from Hirth and Abdel Latif (1980) and West Atlantic data was from Marcovaldi et al. (1999). Regions with significantly different clutch sizes are represented by superscript letters above the bars.

**Fig 4:**
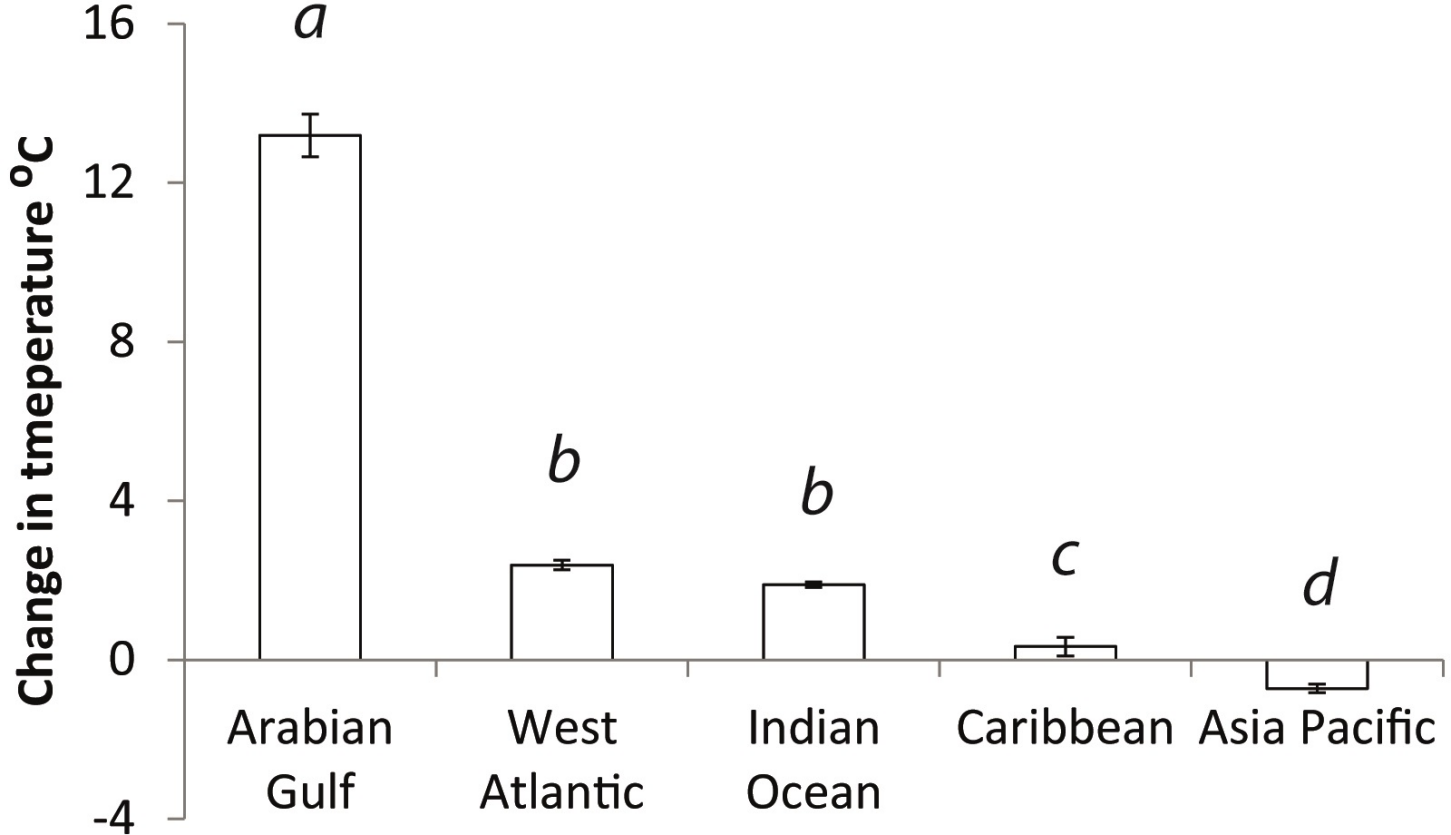
Comparison of the change in air temperatures from the start to end of nesting season in different global regions. Significant difference was found (ANOVA: F_4,86_ = 560.5, p<0.01). Data from the Arabian Gulf was from the present study and Pazira et al. (2015); data from the Caribbean was collected from Richardson et al. (1999) and Kamel and Delacroix (2007); Indian Ocean data was from Mortimer and Bresson (1999); Asia Pacific data was from Hamilton et al. (2015) and West Atlantic data was from Marcovaldi et al. (1999).

**Fig 5:**
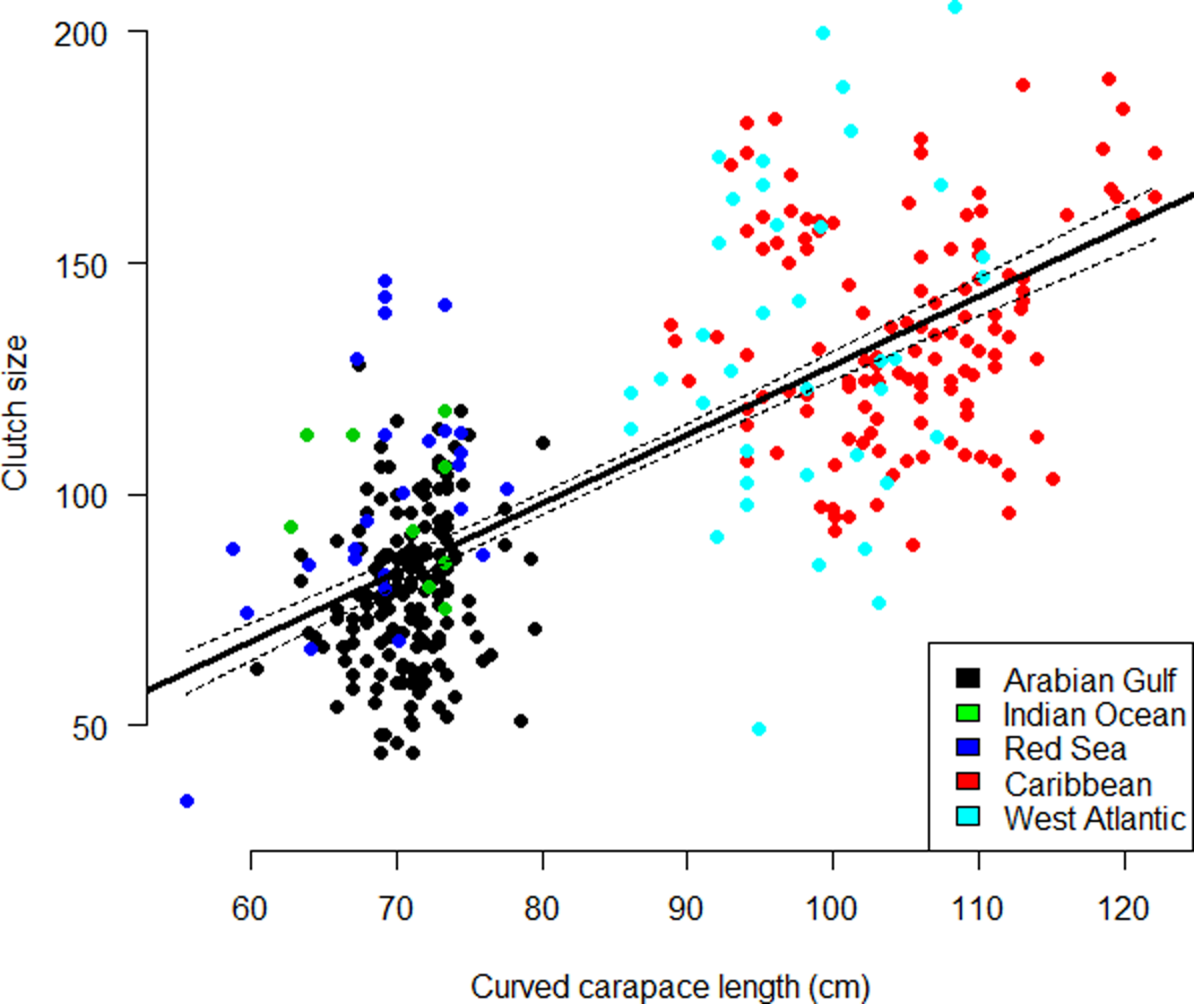
Linear regression of CCL and clutch size using data from different global regions. Significant relationship found (Regression: F_4,368_ = 64.59; R^2^ = 0.60; p<0.01). Solid line represents the mean and dotted lines show 95% confidence intervals. Data for the Arabian Gulf was from the present study and Pazira et al. (2016); raw data from the Caribbean was collected from Xavier et al. (2006); Ross (1980) provided data from the Indian Ocean; Red Sea data was from Hirth and Abdel Latif (1980) and West Atlantic data was from Marcovaldi et al. (1999).

Significant variation in CCL’s between global regions was detected (ANOVA: F_4,470:_ = 989.6; p<0.01, Fig 2). Further post hoc analysis showed Arabian Gulf hawksbills to be significantly smaller than populations already reported from the Caribbean (p<0.01), Asian Pacific (p<0.01) and Indian Ocean (p<0.01) but not from the Red Sea (p = 0.19). Additionally, significant variation in clutch sizes between global regions (ANOVA: F_4,412_ = 156.2; p<0.01, Fig 3) was found. Post hoc analysis showed the Arabian Gulf to have smaller clutches than all other global regions (Caribbean p<0.01, West Atlantic p<0.01 and Red Sea p<0.01) apart from the Indian Ocean (p = 0.08). Change in temperature from the start of the nesting season to the end also showed significant variation with region (ANOVA: F_4,86_ = 560.5, p<0.01, Fig 4). Arabian Gulf air temperatures increased on average 13.2°C (SD±0.26) from the start to end of nesting (raw interpolated data in S4 Table). Tukey analysis showed this change to be significantly greater than other global regions (Asia Pacific p<0.01, Indian Ocean p<0.01, Caribbean p<0.01 and West Atlantic p<0.01). In addition, analysis of already published data in combination with the present study’s showed a significant relationship between CCL and clutch size (Regression: F_4,368_ = 64.59; R^2^ = 0.60; p<0.01; Fig 5) with no interactive effect of region clutch size and CCL (ANCOVA: F_4,368_ = 1.8, p = 0.12).

## Discussion

These results show that Arabian Gulf hawksbill populations display a lower fecundity than other global populations and as a result are more vulnerable than their already endangered counterparts elsewhere in the world. Hawksbills in the Arabian Gulf were significantly smaller and laid fewer eggs per clutch per year. In addition, air temperatures before and after nesting showed a significantly greater increase than other global locations.

It may be argued that hawksbills tagged in this study, represent a younger reproductive population within the Arabian Gulf. However, it is more likely that this CCL is representative of a smaller mature nesting population. Over seven years of data, the size of nesting females did not vary and the largest CCL recorded was 74 cm. This was an individual which had nested on four occasions over a period of six years suggesting she was a mature adult and still only 74 cm. Reproductive females as small as 55 cm Straight Carapace Length (SCL, converts to 57.6 cm CCL) have been recorded in Cuba, suggesting that Arabian Gulf hawksbills are not uniquely small [44]. However, 51–55 cm SCL only represented 1.5% of the total breeding female population in Cuba [44]. Moreover, over 50% reproducing females in Cuba were > 76 cm SCL (converts to 79.6 cm CCL) [44]. Conversely no nesting females > 75 cm CCL have previously been reported in the Arabian Gulf [13,45].

Additionally, hawksbills in the Arabian Gulf lay significantly smaller clutches than other global regions (Fig 3). Though it has not been investigated in hawksbills, clutch size in other sea turtles has previously been linked, in theory, to foraging success and habitat productivity [45]. Variations in clutch size between different sized turtles can be a sign of fluctuating resource availability over its life history [45]. The Arabian Gulf is a semi-enclosed sea where coastal habitats are exposed to extreme summer heat, hyper-salinity and reduced productivity [36]. Moreover, coral reef systems in the Arabian Gulf experience high mortality rates from winter to summer months which has caused a shift in communities to consist of a heat tolerant subset of Indian Ocean corals [24]. Coastal mangroves, in other parts of the world are highly productive outwelling systems, in the Arabian Gulf they act as a nutrient sink from surrounding offshore habitats [46]. This harsh, less productive coastal environment may be a symptom of an extreme marine environment, restricting individual hawksbill growth and subsequently clutch size.

A strong link was found between global populations clutch size and CCL (Fig 5). Correlation between these characteristics in hawksbills from one location has previously been observed [9], however, a combination of global data has explained more variation in clutch size than previously reported. Xavier et al. (2006) found a weak relationship between CCL and fecundity, regression only explained 4% of variance in clutch size. The current study also only found 7% of the variation in clutch size explained by CCL. However, combining global data revealed a much stronger relationship than has previously been observed, explaining 60% of the variation in clutch size (Fig 5). These findings suggest that, in some part due to their reduced size, hawksbills in the Arabian Gulf lay smaller clutches than other regions.

Mean OII found in the present study was comparable to other studies in the Caribbean, Malaysia and the Seychelles which reported a mean OII of 14 to 15 days [2–4,28]. Whilst clutch frequencies in the present study are not conclusive, they are comparable to the mean 1.4 ECF by Pilcher et al. (2014) in the Arabian Gulf, where they used satellite trackers. Despite data from the current study not being extensive enough to accurately estimate interesting intervals and clutch frequencies (not complete coverage and only 7 years of data), it does add data to an under-represented region in hawksbill study. In other regions, clutch frequencies from 1 to 6 per season have been reported and it is well documented that recorded clutch frequency is a function of survey effort [2,3,28,33]. As survey efforts increase so do OCF and ECF [28]. Survey coverage in the present study was relatively low (77%), therefore it is probable that clutch frequencies in the present study have been underestimated. The addition of a correction factor of 23% to account for missed coverage would provide a higher ECF of 1.7 per season. Regardless, this estimate is still lower than estimates reported in the Caribbean (ECF: 2) [28], Brazil (ECF: 4.1) [10] and in the Seychelles (Indian Ocean (ECF: 4) [3]). Raw data of ECF’s presented here may be beneficial to further research and conservationists in the future.

The extreme environment of the Arabian Gulf may also be limiting the length of nesting seasons observed in the region. From seven seasons, the hawksbill nesting period in Qatar was two months, similarly to 3 months reported in western Iran, which may be shorter than other global nesting populations [34,35]. Nesting seasons of 5, 6 months or year round have been reported in Brazil, the Caribbean, and Malaysia respectively [2,5,28]. The shorter nesting season observed in the Arabian Gulf may also be as a result of extreme environmental conditions. Air temperatures in the region have a much greater increase over a shorter period of time than nesting areas elsewhere in the world. Hawksbills within the region nest from the end of spring into the summer months [34,35]. Summer air temperatures and SST regularly exceed 50°C and 30°C respectively. It has also been observed that during summer months, hawksbills in the region leave hotter coastal waters to seek out cooler temperatures in the middle of the Gulf [47]. Perhaps more importantly, air temperatures, which have a significant effect on incubating temperatures [36,48], may be too high in July and August in the Arabian Gulf to successfully incubate clutches. Incubation time and hatching success has been shown to be reduced as ambient temperature increases [49]. In hawksbills, it has been estimated that hatching success is less than 40% if mean incubating temperature is above 33°C and 0% above 33.5°C [50]. A predictive model for sand temperature from air temperature (sand temperature = 0.7154 * air temperature + 9.6023) developed by Hawkes et al (2007) would forecast incubating temperatures >36°C by the end of nesting seasons shows nests in the region are already testing the upper limit of incubating temperatures. The annual period from March to June may be the only suitable window for both adults to be in coastal waters, nest and for eggs to successfully incubate.

Our findings suggest that hawksbill turtles in the Arabian Gulf display a divergent nesting ecology in many aspects relative to other nesting habitats globally [3,4,28,34,35,39]. An extreme environmental setting where air temperatures dramatically change during nesting periods, coupled with the fact that Arabian Gulf hawksbill are smaller and lay smaller clutches per year demonstrates that the region’s population is more vulnerable than those nesting at other rookeries. Moreover, as air temperatures in the Arabian Gulf are predicted to rise into the 21^st^ century [51], the species will only become more vulnerable and at risk from an increasingly extreme environment.

## Supporting information

S1 Table Global dataset. Raw data sets colleated from previously published work. Data includes length of nesting season, CCL, CCW and clutch sizes.
(CSV)

S2 Table Nesting season lengths. Start and end dates of each nesting season in Qatar with beach coverage calculated from the number of nights where technicians were patrolling.
(CSV)

S3 Table Tagging, CCL, CCW and clutch data. Raw data set of all turtles tagged, measured and clutch sizes confirmed in Qatar over the entire study period.
(CSV)

S1 Fig Observed internesting intervals and clutch frequencies. Observed internesting intervals and clutch frequencies for turtles in Qatar over the entire study period.
(TIFF)

S4 Table Interpolated air temperature data. Reported nesting seasons from global studies with start and end months each year. From these dates and locations, air temperatures were interpolated from global gridded data sets from the NOAA National Center for Environmental Prediction.
(CSV)
