# Supplementary figures and images for: Nesting ecology of hawksbill turtles, Eretmochelys imbricata, in an extreme environmental setting
Source: PLoS One. 2018 Sep 7;13(9):e0203257. doi: 10.1371/journal.pone.0203257 (PMC6128527; doi:10.1371/journal.pone.0203257)

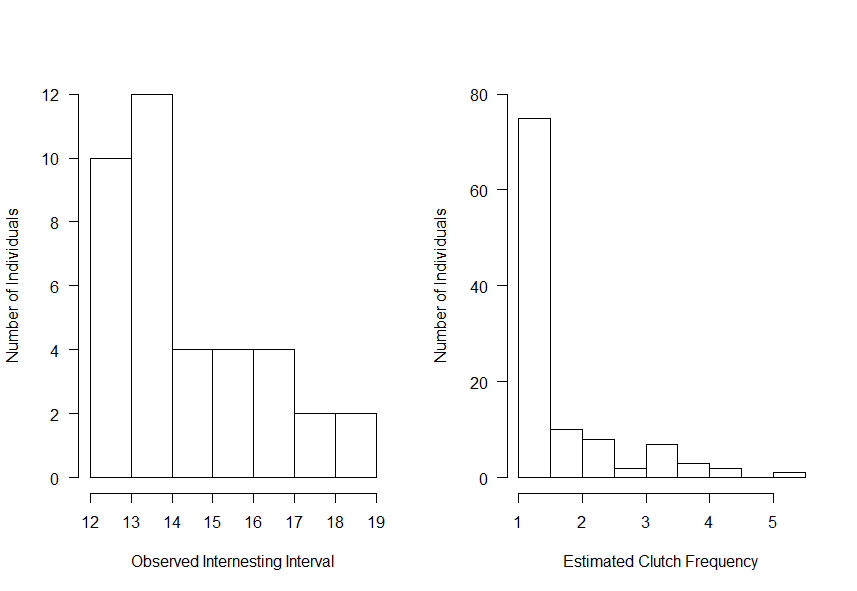

Supplement: S1 Fig — Observed internesting intervals and clutch frequencies for turtles in Qatar over the entire study period. (TIFF) [file pone.0203257.s004.tiff]
